# In vitro synergistic potentials of novel antibacterial combination therapies against *Salmonella enterica* serovar Typhimurium

**DOI:** 10.1186/s12866-020-01810-x

**Published:** 2020-05-14

**Authors:** Md. Akil Hossain, Hae-Chul Park, Kwang-Jick Lee, Sung-Won Park, Seung-Chun Park, JeongWoo Kang

**Affiliations:** 1 Veterinary Drugs & Biologics Division, Animal and Plant Quarantine Agency, Gimcheon-si, 39660 Republic of Korea; 2 Laboratory of Veterinary Pharmacokinetics and Pharmacodynamics, College of Veterinary Medicine, Kyungpook National University, Bukgu, Daegu, 41566 Republic of Korea

**Keywords:** Combination therapy, Critically important antibiotics, Phenolic compounds, Gallic acid, Hamamelitannin, Biofilm

## Abstract

**Background:**

The antibiotics generally used in farm animals are rapidly losing their effectiveness all over the world as bacteria develop antibiotic resistance. Like some other pathogenic bacteria multidrug-resistant strains of *Salmonella enterica serovar* Typhimurium (*S.* Typhimurium) are also frequently found in animals and humans which poses a major public health concern. New strategies are needed to block the development of resistance and to prolong the life of traditional antibiotics. Thus, this study aimed to increase the efficacy of existing antibiotics against *S.* Typhimurium by combining them with opportunistic phenolic compounds gallic acid (GA), epicatechin, epicatechin gallate, epigallocatechin and hamamelitannin. Fractional inhibitory concentration indexes (FICI) of phenolic compound-antibiotic combinations against *S.* Typhimurium were determined. Based on the FICI and clinical importance, 1 combination (GA and ceftiofur) was selected for evaluating its effects on the virulence factors of this bacterium. Viability of *Rattus norvegicus* (IEC-6) cell in presence of this antibacterial combination was evaluated.

**Results:**

Minimum inhibitory concentrations (MICs) of GA, epigallocatechin and hamamelitannin found against different strains of *S.* Typhimurium were 256, (512–1024), and (512–1024) μg/mL, respectively. Synergistic antibacterial effect was obtained from the combination of erythromycin-epicatechin gallate (FICI: 0.50) against *S.* Typhimurium. Moreover, additive effects (FICI: 0.502–0.750) were obtained from 16 combinations against this bacterium. The time-kill assay and ultrastructural morphology showed that GA-ceftiofur combination more efficiently inhibited the growth of *S.* Typhimurium compared to individual antimicrobials. Biofilm viability, and swimming and swarming motilities of *S.* Typhimurium in presence of GA-ceftiofur combination were more competently inhibited than individual antimicrobials. Viabilities of IEC-6 cells were more significantly enhanced by GA-ceftiofur combinations than these antibacterials alone.

**Conclusions:**

This study suggests that GA-ceftiofur combination can be potential medication to treat *S.* Typhimurium-associated diarrhea and prevent *S.* Typhimurium-associated blood-stream infections (e.g.: fever) in farm animals, and ultimately its transmission from animal to human. Further in vivo study to confirm these effects and safety profiles in farm animal should be undertaken for establishing these combinations as medications.

## Background

Livestock (farm animals) are one of the most important and rapidly expanding commercial agricultural sectors worldwide [1]. Infectious agents transmitted between livestock and humans are important to both public health and livestock economics [2]. Infectious diseases cause direct losses to livestock sector through increased mortality and reduced livestock productivity, as well as indirect losses associated with cost of control, loss of trade, decreased market values, and food insecurity [1]. It is estimated that 75% of emerging human infectious diseases are zoonotic origins, with livestock serving as important reservoirs of infection [3].

*Salmonella enterica Serovar* Typhimurium (*S*. Typhimurium) is likely to acquire and retain antimicrobial resistance genes following exposure to antimicrobial agents in animals, and this is the most frequently identified serovar during human gastrointestinal disease outbreaks in different parts of the world [4–6]. A large number of *S*. Typhimurium isolates from various species of animals and humans are multidrug-resistant and pose a major public health concern [7].

The efficacies of currently available antibiotics are continuously reducing due to the emergence of multidrug-resistant strains [8–11]. Hence, alternative intervention measures to minimize the microbial load in livestock animals are urgently needed, which will ultimately reduce the public health risks. One of the novel strategies is the combination of antibacterials, which may increase the efficacies of existing antibiotics against multidrug-resistant bacteria like *S.* Typhimurium [12]. Phenolic compound (methyl gallate and pyrogallol)-containing *Nymphaea tetragona* 50% methanol extract (NTME) was evident in our earlier studies for synergistic antibacterial and quorum sensing (QS) inhibition effects [13, 14]. The phenolic compound gallic acid (GA) demonstrated the potential to inhibit biofilms of *S. mutans* [15]. Recently, we also reported that methyl gallate, a GA derivative, can efficiently interfere with the QS regulatory pathways of *P. aeruginosa*, and inhibit the adhesion, invasion and intracellular survival of *S.* Typhimurium [16, 17]. These properties of bacteria are known to have significant roles in increasing pathogenicity and antimicrobial resistance [18].

GA derivatives contain a large number of hydroxyls, which can form protonic and ionic bonds and combine with many biological proteins, such as enzymes, carriers, ion channels and receptors, deactivating them, and consequently exhibiting bacterial inhibition. Additionally, many phenols can non-specifically affect molecular targets of microorganism [19]. These observations and findings of our previous studies initiate the speculation that GA derivatives may also increase the efficacies of existing antibiotics. Thus, we intended in this study to evaluate the antibacterial potentials of GA and its 4 derivatives individually; and in combination with 8 commercially available antibiotics against *S.* Typhimurium. Additionally, the effects of these selected antibacterial combinations against virulence factors, including biofilm formation and motility were determined. Finally, the effects of GA alone and in combination with commercial antibiotics on the viability of *Rattus norvegicus* small intestine (IEC-6) cells were investigated.

## Results

### Antibacterial activities of commercial antibiotics and phenolic compounds

Antibacterial activities of several commercially available antibiotics and phenolic compounds were evaluated against quality control (QC) as well as clinical strains of *S.* Typhimurium. The minimum inhibitory concentrations (MICs) of different commercial antibiotics (amoxicillin, ampicillin, cefotaxime, ceftiofur, erythromycin, florfenicol, marbofloxacin, norfloxacin, penicillin G and theamphenicol) against QC strain of *S.* Typhimurium ranged from 0.062–128 μg/mL. In contrast, the MICs of these commercial antibiotics against the clinical isolates of *S.* Typhimurium ranged from 0.25 to ≥1024 μg/mL. The results in Table 1 clearly demonstrate that the MICs of almost all of these commercial antibiotics against the clinical isolates were increased by several folds, which indicates that resistance has developed in these clinical strains [20–24]. The MICs of phenolic compounds (epicatechin, epicatechin gallate, epigallocatechin, GA and hamamelitannin) against the QC strains and clinical isolates of *S.* Typhimurium ranged from 256 to ≥1024 μg/mL, with GA being the most potent among all these compounds.

**Table 1:** Minimum inhibition concentration (MIC) of commercial antibiotics and phenolic compounds against different strains of *Salmonella enterica serovar* Typhimurium; and antibacterial sensitivity profiles of those strains against some selected commercially available antibiotics

Antimicrobials	Strain ID					
	ATCC 14028	V08-S-HA-06-(170)	V15-S-HA-02-(210)	SAL 109	SAL 202	SAL 224
Amoxicillin (μg/mL)	< 0.5 (S)	256 (R)	1 (S)	256 (R)	256 (R)	256 (R)
Ampicillin (μg/mL)	1 (S)	512 (R)	512 (R)	512 (R)	512 (R)	512 (R)
Cefotaxime (μg/mL)	≤ 2 (S)	≤ 2 (S)	≤ 2 (S)	512 (R)	128 (R)	128 (R)
Ceftiofur (μg/mL)	< 1 (S)	< 1 (S)	1 (S)	128 (R)	64 (R)	64 (R)
Erythromycin (μg/mL)	128 (R)	32 (R)	16 (R)	512 (R)	1024 (R)	512 (R)
Florfenicol (μg/mL)	8 (S)	8 (S)	4 (S)	64 (R)	32 (R)	64 (R)
Marbofloxacin (μg/mL)	0.062 (S)	0.25 (S)	0.25 (S)	2 (I)	2 (I)	4 (I)
Norfloxacin (μg/mL)	0.25 (S)	4 (R)	0.5 (S)	4 (R)	16 (R)	8 (R)
Penicillin G (μg/mL)	8 (S)	1024 (R)	32 (R)	> 1024 (R)	> 1024 (R)	> 1024 (R)
Thiamphenicol (μg/mL)	128 (R)	64 (R)	128 (R)	256 (R)	512 (R)	512 (R)
Epicatechin (μg/mL)	> 1024	> 1024	> 1024	> 1024	> 1024	> 1024
Epicatechin gallate (μg/mL)	> 512	512	> 512	> 512	> 512	> 512
Epigallocatechin (μg/mL)	1024	512	512	512	512	512
Gallic acid (μg/mL)	256	256	256	256	256	256
Hamamelitannin (μg/mL)	512	512	1024	1024	1024	1024

S, susceptible; I, intermediate resistance; R, resistant. Sensitivity statuses of these strains are interpreted based on the MIC values of these tested antibiotics and their break-point MIC values mentioned in different guidelines [20–24]

### Combination interactions of commercial antibiotics with phenolic compounds

Checkerboard microdilution assay was performed to evaluate combination interactions of commercial antibiotics with phenolic compounds for bacterial inhibition. The results of the combined activities as fractional inhibitory concentration indexes (FICI) are presented in Table 2. The combination of erythromycin and epicatechin gallate against *S.* Typhimurium showed synergistic antibacterial effects (FICI: 0.50). Moreover, additive effects (FICI: 0.502–0.750) were obtained from 16 combinations, and indifferent effects (FICI: 1.001–1.016) were obtained from 23 combinations against *S.* Typhimurium. Antagonistic effect was not found from any of the combinations. GA-ceftiofur combination that had additive antibacterial effect against *S.* Typhimurium was selected for further study depending on both the clinical and commercial importance.

**Table 2:** Combination interaction of phenolic compounds with commercial antibiotics against *Salmonella enterica serovar* Typhimurium (ATCC14028)

Drug Combinations	FIC Index (X)
Galic acid + Ampicillin	0.625 (A)
Galic acid + Amoxicillin	1.004 (I)
Galic acid + Ceftiofur	0.563 (A)
Galic acid + Penicillin G	1.016 (I)
Galic acid + Cefotaxime	1.004 (I)
Galic acid + Erythromycin	1.001 (I)
Galic acid + Thiamphenicol	0.625 (A)
Galic acid + Marbofloxacin	1.002 (I)
Hamamelitannin + Ampicillin	1.002 (I)
Hamamelitannin + Amoxicillin	1.001 (I)
Hamamelitannin + Ceftiofur	0.625 (A)
Hamamelitannin + Penicillin G	1.004 (I)
Hamamelitannin + Cefotaxime	0.563 (A)
Hamamelitannin + Erythromycin	1.001 (I)
Hamamelitannin + Thiamphenicol	1.002 (I)
Hamamelitannin + Marbofloxacin	0.563 (A)
Epicatechin + Ampicillin	0.750 (A)
Epicatechin + Amoxicillin	1.001 (I)
Epicatechin + Ceftiofur	0.625 (A)
Epicatechin + Penicillin G	1.004 (I)
Epicatechin + Cefotaxime	0.750 (A)
Epicatechin + Erythromycin	1.016 (I)
Epicatechin + Thiamphenicol	1.016 (I)
Epicatechin + Marbofloxacin	1.004 (I)
Epicatechin gallate + Ampicillin	0.563 (A)
Epicatechin gallate + Amoxicillin	1.002 (I)
Epicatechin gallate + Ceftiofur	1.001 (I)
Epicatechin gallate + Penicillin G	0.502 (A)
Epicatechin gallate + Cefotaxime	1.004 (I)
Epicatechin gallate + Erythromycin	0.500 (S)
Epicatechin gallate + Thiamphenicol	0.504 (A)
Epicatechin gallate + Marbofloxacin	1.008 (I)
Epigallocatechin + Ampicillin	1.001 (I)
Epigallocatechin + Amoxicillin	1.004 (I)
Epigallocatechin + Ceftiofur	0.625 (A)
Epigallocatechin + Penicillin G	1.008 (I)
Epigallocatechin + Cefotaxime	0.502 (A)
Epigallocatechin + Erythromycin	0.516 (A)
Epigallocatechin + Thiamphenicol	1.004 (I)
Epigallocatechin + Marbofloxacin	0.502 (A)

FIC, fractional inhibitory concentration; Synergy, X ≤ 0.5; Additive, 0.5 < X ≤ 1; Indifferent, 1 < X ≤ 2; Antagonist, X > 2; (A), (I) and (S) stand for Additive, Indifferent and Synergy, respectively

### Effects of combination drugs on time- and concentration-dependent inhibition

**Fig. 1:**
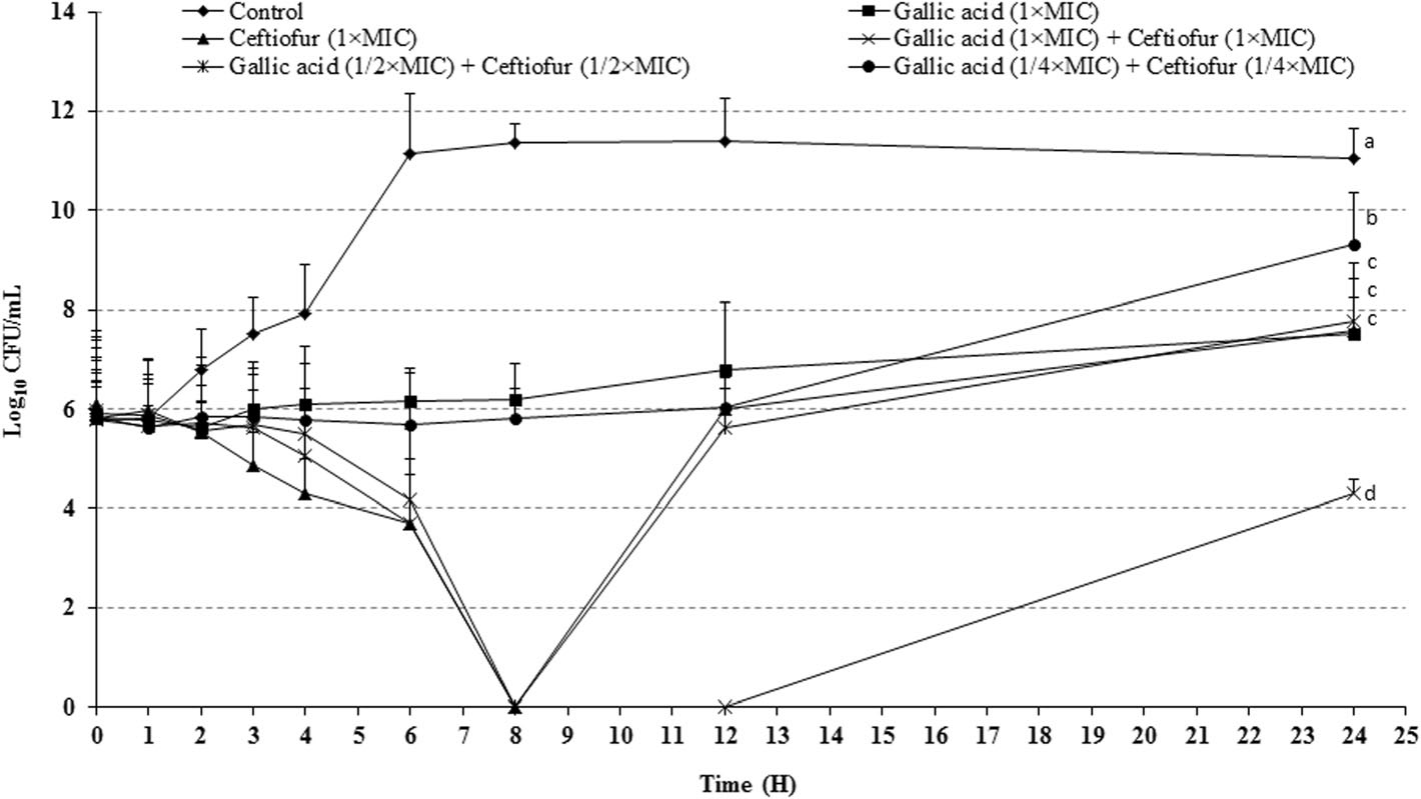
Time-kill curves of *Salmonella enterica serovar* Typhimurium (ATCC14028) in presence of gallic acid-ceftiofur combination. MIC, minimum inhibitory concentration. Different superscript letters (a, b, c, and d) indicate statistical significance (*p* < 0.05) among different sample groups. Results are interpreted from 3 independent experiments

The inhibitory effects of the combination antibacterials on bacterial growth rates over time are presented in Fig. 1. The growth rate of *S.* Typhimurium was approximately the same when a 1 × MIC concentration of GA was supplemented into the culture, and the cell density increased by approximately 1-fold at 24 h compared to the density at the time of inoculation. After 24 h, the cell density of the bacterium in the presence of 1 × MIC ceftiofur reached a level which is 1-fold higher than its initial density. In contrast, the cell number in the drug-free control culture increased by approximately 5-fold within 6 h, and the same cell density was sustained out to 24 h of incubation. Treatment of *S.* Typhimurium for 24 h with the antibacterial combination (1 × MIC of both ceftiofur and GA) showed a 2-fold less cell density than its initial density. The combination of ceftiofur (½ × MIC) and GA (½ × MIC) prevented this increase of growth in such a way that the final cell density at 24 h were almost the same as their initial cell density. Moreover, the ¼ × MIC of both drugs together inhibited the growth by approximately 2-fold, which demonstrated the potential of this combination drug for bacterial inhibition.

### Effects of combination drugs on the morphology of bacterial cells

**Fig. 2:**
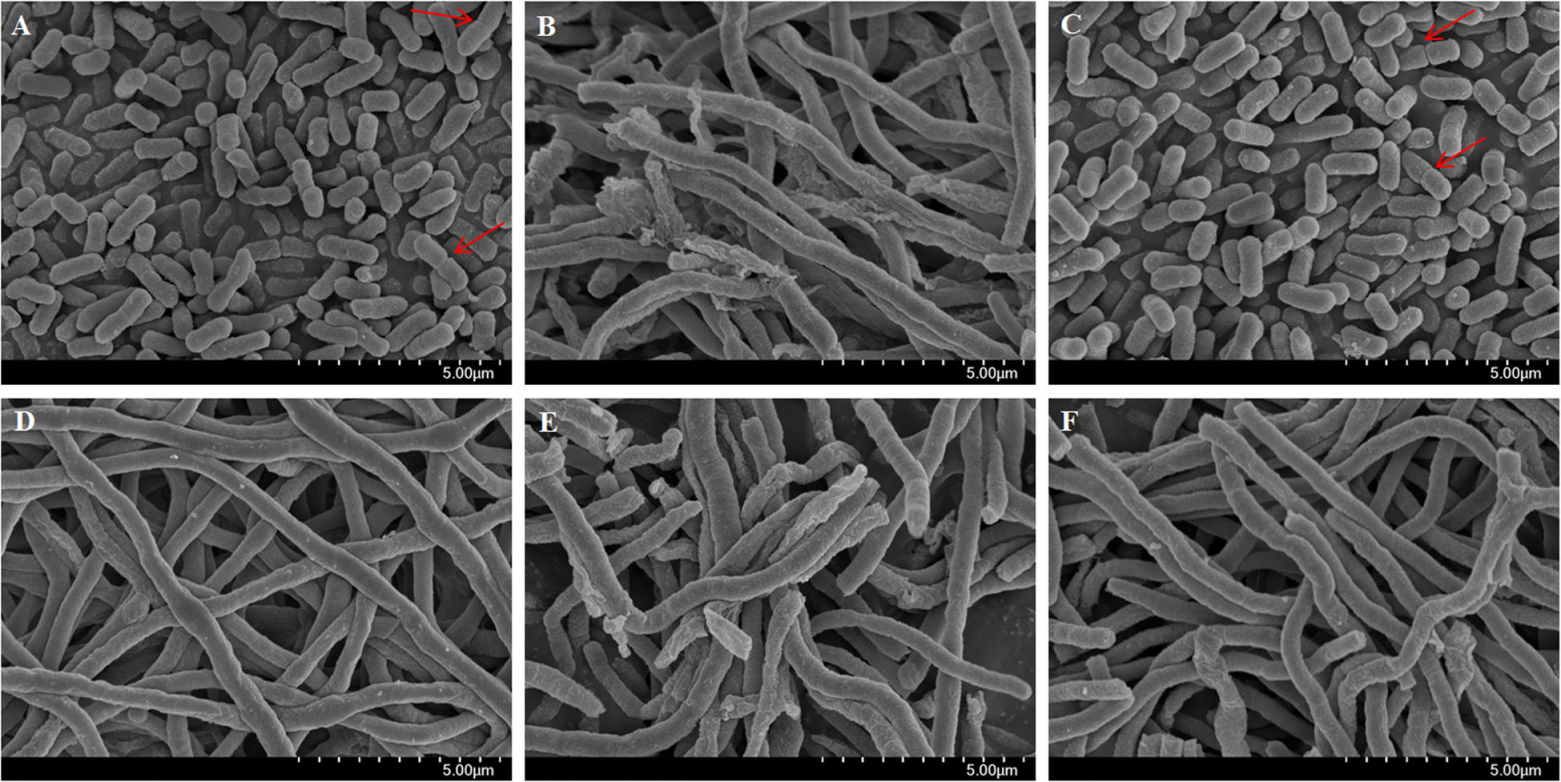
Effect of gallic acid-ceftiofur combination on the ultrastructure morphology of *Salmonella enterica serovar* Typhimurium (ATCC14028) cells. Representative images of bacterial cells captured by scanning electron microscope after treated with antibacterial combination. *Salmonella enterica serovar* Typhimurium cells treated with (A) no drug, (B) ceftiofur (1 × MIC), (C) gallic acid (1 × MIC), (D) ceftiofur (1 × MIC) + gallic acid (1 × MIC), (E) ceftiofur (½ × MIC) + gallic acid (½ × MIC), and (F) ceftiofur (¼ × MIC) + gallic acid (¼ × MIC). MIC, minimum inhibitory concentration. Arrows indicate binary fission

The ultrastructural morphologies of *S.* Typhimurium cells treated with GA-ceftiofur combination were studied to assess whether the combination drugs had any impact on the cellular architecture. The representative scanning electron microscope (SEM) images of GA- and ceftiofur-treated *S.* Typhimurium cells are shown in Fig. 2. The SEM images revealed that untreated and GA (1 × MIC)-treated *S.* Typhimurium cells had rod-like shape, and they were separated with perfect symmetry. In addition, binary fission of the bacteria was evident in the SEM images (Fig. 2a and c). The cells treated with ceftiofur alone or in combination with GA were found in a long rope-like shape, and no binary fission was evident, which is completely different from control cells. None of the cells were pitted, deformed or broken and the antibacterials had no effect on the cell wall or cytoplasmic membrane of the bacteria.

### Effects of combination drugs on biofilm inhibition and viability

**Fig. 3:**
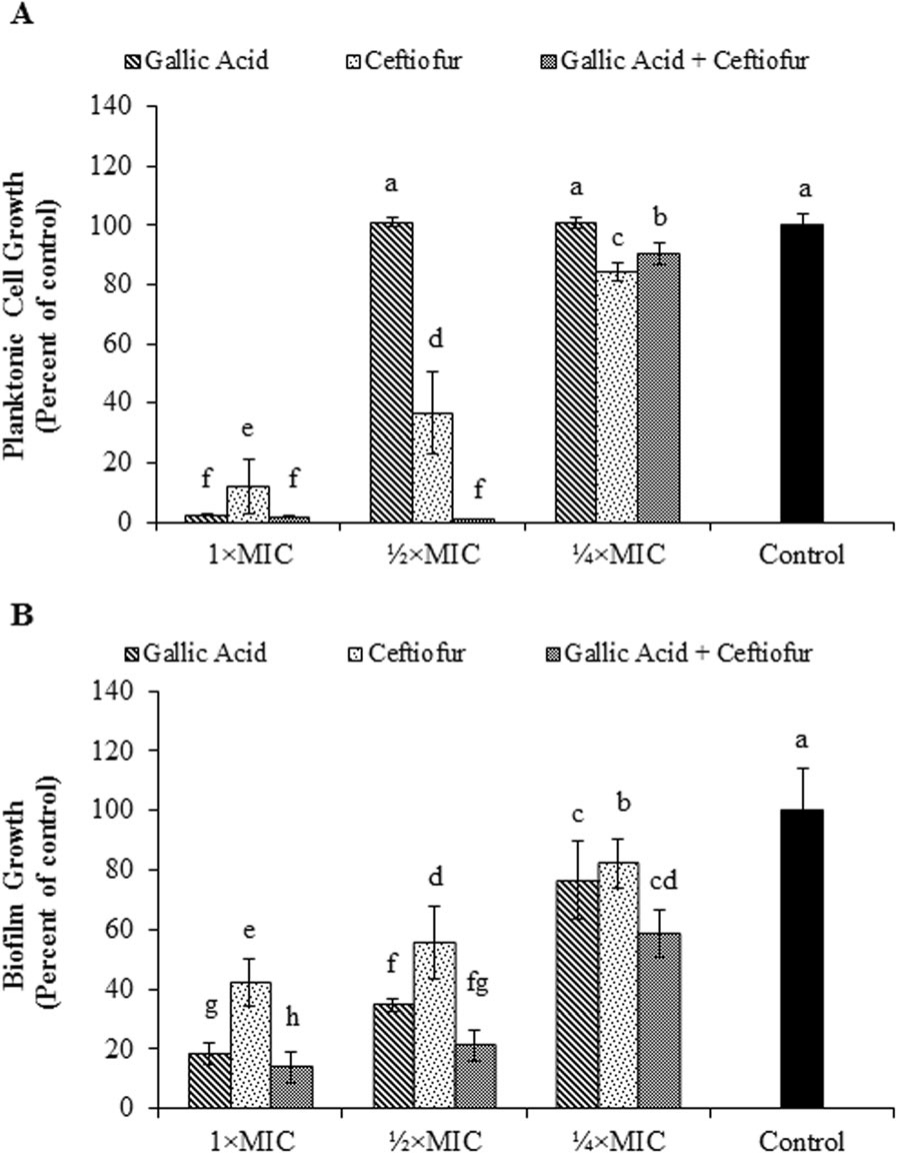
Effect of gallic acid-ceftiofur combination on (A) planktonic and (B) biofilm cells of *Salmonella enterica serovar* Typhimurium (ATCC14028). MIC: minimum inhibitory concentration. Different superscript letters indicate statistical significance (*p* < 0.05). Results are interpreted from 3 independent experiments

*S.* Typhimurium biofilm formation in the presence of GA-ceftiofur combination was evaluated. The effects of the combination antibacterials on the growth of planktonic and biofilm cells of *S.* Typhimurium are shown in Fig. 3. The inhibition of both planktonic and biofilm cells of this bacterium was more induced by the combination of GA and ceftiofur than by the individual drugs in most cases. Even, the ¼ × MIC of ceftiofur could significantly inhibit *S.* Typhimurium biofilm formation when a lower amount of GA was applied together with this commercial antibiotic.

**Fig. 4:**
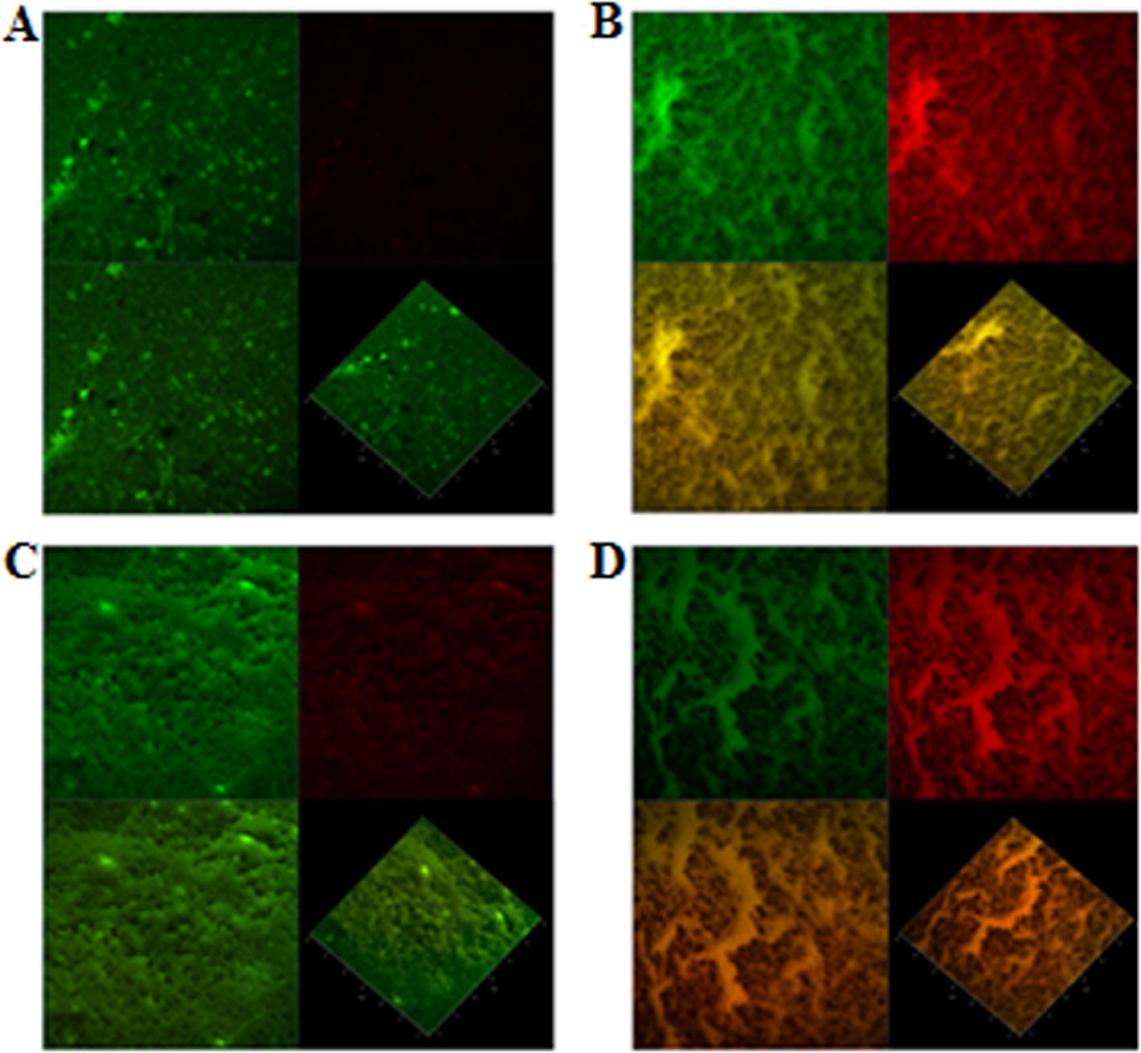
Effect of gallic acid-ceftiofur combination on the viability of cultured biofilm. The confocal laser scanning microscope images of BacLight LIVE/DEAD stained biofilms of *Salmonella enterica serovar* Typhimurium (ATCC14028) treated with (**A**) no drug, (**B**) gallic acid (½ × MIC), (**C**) ceftiofur (½ × MIC), and (**D**) gallic acid (½ × MIC) + ceftiofur (½ × MIC). The viability of the biofilm cells was assessed using BacLight LIVE/DEAD stain (green: live cells, red: dead cells). Four different segments are present in each of the images (**A**, **B**, **C** and **D**) where, the top left segment shows only SYTO9-stained cells, the top right segment displays only propidium iodide-stained cells, and below left and below right segments show merged images of both SYTO9-stained cells and propidium iodide-stained cells from an individual sample. In each image, the segment at below right side shows three dimensional and other three segments show two dimensional images

**Fig. 5:**
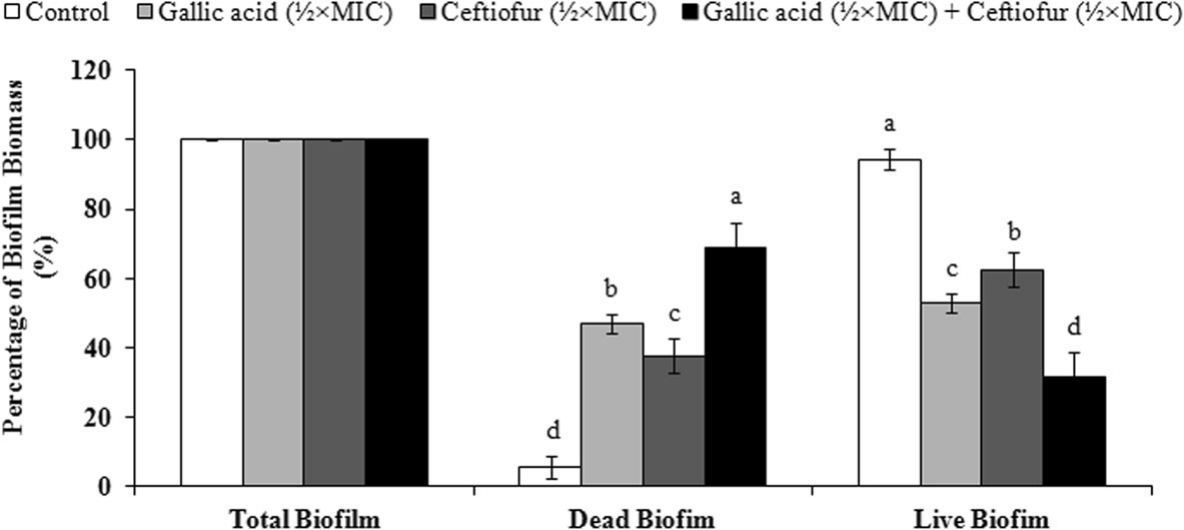
Percentage of biofilm biomass of *Salmonella enterica serovar* Typhimurium (ATCC14028) in presence of gallic acid-ceftiofur combination. Biomasses of total biofilm in each test group were considered 100%, and calculated the biomass percentages of dead biofilm (propidium iodide-stained) and live biofilm (SYTO9-stained). Results are interpreted from 3 independent experiments and shown as (mean ± SD). Different superscript letters indicate significant differences (*p* < 0.05) among test groups within individual type of biofilm biomass. MIC, minimum inhibitory concentration

The viability of *S.* Typhimurium biofilm in the presence of GA-ceftiofur combination was determined by staining the biofilms with BacLight live/dead stain and imaging with a confocal laser scanning microscope (CLSM). The reduction in biofilm viability with the combination antibacterial treatment is demonstrated by the results obtained with the CLSM. Figures 4 and 5 are respectively showing the CLSM images and biomass percentages of *S.* Typhimurium biofilms formed on a glass surface and treated with or without GA-ceftiofur combination. The confocal micrograph of 48 h *S.* Typhimurium biofilms treated with combination antibacterials for 24 h displays a higher proportion of dead cells (cells that are stained red) than those observed in the untreated control. It is clearly visible from the biomass of total biofilm, live biofilm (SYTO9-stained biofilm) and dead biofilm (propidium iodide-stained biofilm) as presented in Fig. 5 that almost all the cells were alive in the control biofilm, while a remarkable number of cells were found dead after treating with antibacterials. Percentage of dead biofilm biomass after treating with GA-ceftiofur combination (68.66%) was significantly higher than those obtained by treating with individual antibacterials (37.57–47.01)%.

### Effects of combination drugs on the motility of bacterial cells

**Fig. 6:**
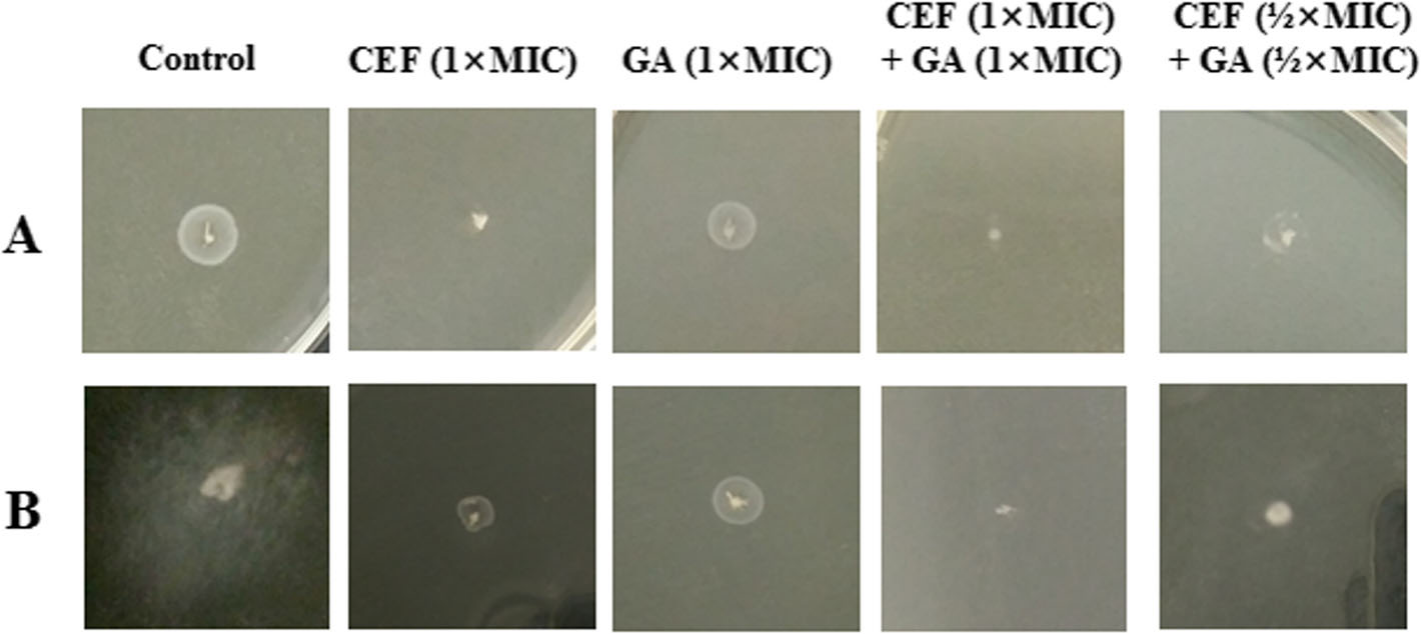
Representative images of swarm (panel A) and swim (panel B) zones of *Salmonella enterica serovar* Typhimurium (ATCC14028) treated with ceftiofur and gallic acid, and ceftiofur-gallic acid combination. CEF, ceftiofur; GA, gallic acid; MIC, minimum inhibitory concentration

The effects of GA-ceftiofur combination on the swimming and swarming motilities of *S.* Typhimurium were evaluated. Representative photographs of drug-treated and non-treated swim and swarm plates are displayed in Fig. 6. Table 3 shows the diameters of the swim and swarm zones. The results demonstrated that the swimming and swarming motilities of *S.* Typhimurium were noticeably inhibited by the GA-ceftiofur combination. Moreover, the combination of GA and ceftiofur showed better inhibitions of swimming and swarming motilities at sub-MIC concentrations compared to their individual effects at 1 × MIC.

**Table 3:** Effect of ceftiofur-gallic acid combination on the swimming and swarming motilities of *Salmonella enterica serovar* Typhimurium (ATCC14028)

Treatment Groups	Swarming (mm) (mean ± SD)	Swimming (mm) (mean ± SD)
Control	9.67 ± 2.08^a^	26.67 ± 2.52^a^
Ceftiofur (1 × MIC)	2.00 ± 1.00^d^	5.33 ± 2.31^c^
Gallic acid (1 × MIC)	6.33 ± 1.15^b^	7.00 ± 1.00^b^
Ceftiofur (1 × MIC) + Gallic acid (1 × MIC)	1.33 ± 0.58^e^	0.00 ± 0.00
Ceftiofur (½ × MIC) + Gallic acid (½ × MIC)	3.33 ± 1.15^c^	2.33 ± 1.53^d^

MIC: minimum inhibitory concentration. Different superscript letters (a, b, c, d and e) indicate statistical significance (*p* < 0.05) among different sample groups. Results are interpreted from 3 independent experiments

### Effects of GA-ceftiofur combination on the viability of IEC-6 cells

**Fig. 7:**
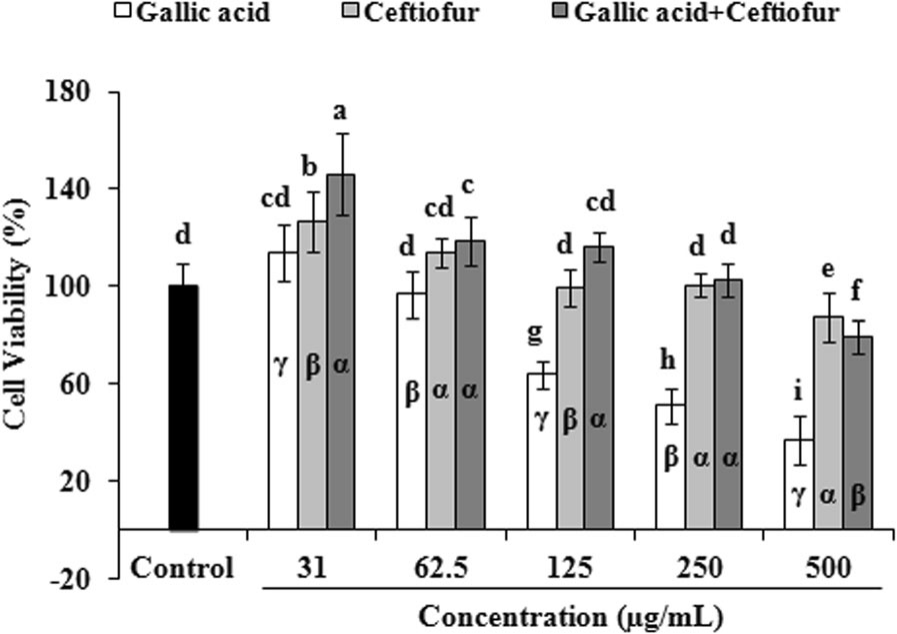
Individual and collective effects of gallic acid and ceftiofur on the viability of *Rattus norvegicus* small intestine (IEC-6) cells. Data represent the mean ± SD of triple assays. Different letters on bars indicate significant differences (*p* < 0.05) compared to drug-free control. Different symbols inside bars indicate significant differences (*p* < 0.05) within same concentration groups

The effects of GA and ceftiofur alone and their combination on the viability of *Rattus norvegicus* (IEC-6) cells are shown in Fig. 7 and Additional file 1. The viabilities of IEC-6 cells were not significantly affected when they were treated only with GA (≤ 62.5 μg/mL). Similarly, ceftiofur (≤ 125 μg/mL) alone had no impact on the viability of IEC-6 cells. Combination of GA (500 μg/mL) and ceftiofur (500 μg/mL) induced the viability of IEC-6 cells than the viability obtained from the treatment of GA (500 μg/mL) alone, but this combination reduced the viability compared to ceftiofur (500 μg/mL) could alone. Combination of GA (250 μg/mL) and ceftiofur (250 μg/mL) did not affect the viability of IEC-6 cells whereas the GA (250 μg/mL) alone significantly interfered with the viability of IEC-6 cells. The inhibitory concentration 50% (IC_50_) of GA in IEC-6 cells was 564.55 μM. The IC_50_ value of GA was more in presence of ceftiofur than the IC_50_ value of GA alone (in absence of ceftiofur). In contrast, IC_50_ value of ceftiofur was less in presence of GA than the IC_50_ value of ceftiofur alone (in absence of GA).

## Discussion

The development of alternative antimicrobial drugs is urgently needed to combat infectious diseases associated with resistant pathogens [25, 26]. The in vitro activities of these tested phenolic compounds against resistant strains of *S.* Typhimurium (Table 1) reflect that some of these compounds could be good candidates to minimize the development of bacterial resistance and to ensure clinical treatment of *S.* Typhimurium infections in farm animals. In the present study, the MIC results demonstrated the antibacterial activities of the phenolic compounds against all the tested strains of *S.* Typhimurium, which have been shown to be resistant to one to nine out of ten currently available antibiotics (Table 1). The potentials of these phenolic compounds were further explored through their combined interactions with commercial antibiotics, where epicatechin gallate possessed synergistic effects with erythromycin against *S.* Typhimurium. The time- and concentration-dependent inhibition assay also exposed that GA-ceftiofur combination more effectively inhibited the growth of *S.* Typhimurium than these antibacterials could alone. Furthermore, the combination of GA and ceftiofur demonstrated improved inhibition of biofilm formation and motility in *S.* Typhimurium.

The MICs of the 5 phenolic compounds (epicatechin, epicatechin gallate, epigallocatechin, GA and hamamelitannin) were investigated against 1 QC strain and 5 clinical strains of *S.* Typhimurium. It has been reported that GA can restrain the growth of many bacteria, including methicillin-sensitive *S. aureus*, MRSA, *E. coli*, *P. aeruginosa*, and *Salmonella typhi* [27]. The results in Table 1 indicate that GA possessed the strongest antibacterial activity among these phenolic compounds, followed by epigallocatechin, hamamelitannin, epicatechin gallate and epicatechin. The MIC values of GA against *S.* Typhimurium (256 μg/mL) in this study were lower than those reported previously (2500 μg/mL) [27]. The mean MIC of plant-derived epigallocatechin against *S.* Typhimurium was reported to be 572 ± 186 μg/mL [28]. The MIC values of pure epigallocatechin against *S.* Typhimurium (512 μg/mL) in our study were lower than the previously reported MIC values [28]. These lower MIC values of pure GA and epigallocatechin against *S.* Typhimurium in our study compared to the MIC values of GA and plant-derived epigallocatechin in the previous studies might be because of the purity of these compounds used. However, the MIC values of plant-derived epigallocatechin against *S.* Typhimurium were comparable with the results of our study. Likewise, the MICs of epicatechin against *S.* Typhimurium were >  1024 μg/mL, which demonstrates the similarity between our results and previously published results [27].

It is always recommended to treat bacterial infections with a combination of antimicrobial agents to prevent the development of drug resistance and to improve efficacy. Drug combinations with synergistic interactions are generally considered to be more effective and, therefore, preferable [29]. Incidentally, synergistic effect (FICI: 0.50) was obtained from the combination of erythromycin and epicatechin gallate against *S.* Typhimurium. Moreover, additive effects (FICI: 0.502–0.750) were obtained from 16 combinations against this bacterium. The rest of the combinations had indifferent effects against this bacterium. Excellent in vitro activity combined with the synergistic effects with other antibacterial drugs underscores the potential utility of these phenolic compounds for the treatment of *S.* Typhimurium-associated infections in farm animals. The combination of GA and ceftiofur which has additive antibacterial effect against *S.* Typhimurium was selected for further studies depending on both the clinical and commercial importance.

Time-kill assays are useful for the evaluation of the pharmacodynamic characteristics of new antimicrobial agents and to determine whether the effects of antibacterials are bacteriostatic or bactericidal [30]. According to our results, GA alone or in combination with ceftiofur show bacteriostatic activity against the tested bacteria, as a reduction ≥99.9% of the inoculum was not observed compared to the growth control. At the end of the incubation period (24 h), a greater than 3-fold reduction in the inoculum concentration with ½ × MIC of GA and ceftiofur and an approximately 7-fold lower inoculums concentration with 1 × MIC of GA and ceftiofur were achieved in contrast to the control. After the incubation period, the inoculum concentrations were reduced by several-fold compared to the control for the bacteria treated with GA alone or in combination with ceftiofur, indicating that these phenolic compounds alone and in combination with ceftiofur has bacteriostatic effects.

In investigating antibacterial effects, it is also essential to evaluate changes in the bacterial cell morphology, membrane permeability and integrity, and surface characteristics [31, 32]. The physiological and morphological changes in *S.* Typhimurium were observed by SEM after treatment with GA alone and in combination with ceftiofur. The results showed a direct effect of the combination antibacterials on the tested pathogens. The treated bacterial cells showed obvious morphological changes compared to untreated cells. Almost all treated bacterial cells were found as long and rope-like, with no binary fission, which is completely different from the untreated control cells. These observations indicate that the GA-ceftiofur combination has a major effect on the bacterial cell division. To exhibit this effect, drug combination possibly induced the expression of SOS operon by damaging DNA or by blocking DNA replication, and/or may inhibited or mutated penicillin-binding proteins (PBP) [33–35].

*S.* Typhimurium biofilm formation in the presence of the GA-ceftiofur combination was evaluated. The inhibition of both planktonic and biofilm cells of this bacterium was induced by the combination drugs more than the individual drugs in most cases. The surviving and dead biofilm populations in the presence of the combination antibacterials were determined by imaging the BacLight live/dead-stained biofilm by CLSM. In this study, 2 laser systems in CLSM were used. Green fluorescent cells can be seen only when SYTO9-stained cell scanning channel is open, and conversely red fluorescent cells can be seen only when propidium iodide-stained cell scanning channel is open. When both channels are open at a time, the system scans both green fluorescent cells and red fluorescent cells, and shows a mixed color (yellowish) depending on the proportion of dead and live cells in that particular sample [36, 37]. Some biofilm cells in our CLSM images are yellow or yellowish color which is justified based on this discussion. The results revealed that the GA-ceftiofur combination more efficiently inhibit the biofilm than these antibacterials individually can. The large effect of the GA-ceftiofur combination against the biofilm cells of *S.* Typhimurium might be due to the small molecular size of GA (170.12 g/mol), which easily penetrates into the biofilm, and subsequently cause detachment of cells and thus the biofilm cells become more exposed and susceptible.

Motility is one of the pathogenic phenotypes of bacteria that contribute to the migration and dispersion of bacteria and their escape from the host immune response [38]. Flagella are known to be involved in swimming motility and play a role in biofilm formation, as well as swarming motility [28]. Recent reports mentioned that, similar to biofilms, swarming cells also show a higher degree of resistance to a variety of antibiotics [39, 40]. In this study, we investigated the ability of the GA-ceftiofur combination to inhibit the swarming and swimming activities of *S.* Typhimurium. The results (Table 3) showed significant inhibition of swimming and swarming motilities with the addition of GA-ceftiofur combination. The lack of swimming and swarming motilities in the presence of the combination antibacterials suggest that these agents might have some effects on flagella-related processes, namely, flagella biosynthesis, rotation, and chemotaxis, which may lead to decreased swimming and swarming activities.

The evaluation of the safety/toxicity profiles of any drug is desirable and an essential part of the investigation of the pharmacological effects. Virtually all organs and tissues are exposed, once the ingested drugs cross the intestinal wall [41]. Many studies have demonstrated that insignificant amounts of orally administered phenolic compounds such as GA are absorbed through the gastrointestinal tract due to its low membrane permeability and poor water solubility [42, 43]. Therefore, the pharmacological or toxicological effects of these phenolic compounds can be largely explained in terms of their local effects. Moreover, this study found additive antibacterial and virulence inhibition effect by the phenolic compound GA against enteric bacteria (*S.* Typhimurium). Likewise, these antibacterial combinations may also be applicable in the eradication of invasive enteric pathogens in the gastrointestinal tract prior to mucosal penetration. Administration of drug through oral route can be more logical to treat this enteric bacterium. Thus, oral administration of this phenolic compound can exert its antibacterial effects within the gastrointestinal lumen and will be unable to reach in the systemic circulation as well as other vital organs. Based on this explanation, oral administration of this compound will have no chance to harm other organs except the gastrointestinal tract. Therefore, investigating the viability of intestinal cell is the particular interest of this study.

Hence, the cytotoxic effects of GA alone and in combination with ceftiofur were investigated in IEC-6 cell lines. Combination of GA and ceftiofur with their highest tested concentration showed increased viability of IEC-6 cells than the viability retained by GA alone. Combining GA with ceftiofur induced the IC_50_ of GA in IEC-6 cells. The effects of GA on cell viability were evaluated previously with different cell lines and found to have cytoprotective effects or no adverse effects in cell viability [44, 45]. Thus, the effects of GA-ceftiofur combination on cell viability obtained in this study are logical and reliable.

## Conclusions

Together with all the promising in vitro assay findings, it is concluded that gallic acid alone, and in combination with ceftiofur can be promising, potent and novel candidate to eradicate pathogenic *S.* Typhimurium*.* The effect of gallic acid is bacteriostatic, and the use of gallic acid-ceftiofur combination can more effectively interfere with the biofilms of *S.* Typhimurium than the antibiotic alone, which is crucial to develop new antibacterials and/or improve the efficacy of existing antibacterials for reducing the pathogenicity associated with this bacterium. This study also suggest that gallic acid-ceftiofur combination can be potential medication to treat *S.* Typhimurium-associated diarrhea and prevent *S.* Typhimurium-associated blood-stream infections (e.g.: fever) in farm animals, and ultimately its transmission from animal to human. Further in vivo studies are recommended to confirm their efficacy and safety in farm animal for enhancing their safe and effective utilization as medications.

## Methods

### Chemicals, reagents and bacterial strains

Luria-bertani (LB), mueller hinton agar (MHA), mueller hinton broth (MHB); cation-adjusted mueller hinton broth (CA-MHB), nutrient broth (NB), trypticase soy broth (TSB) and agar were purchased from Becton Dickinson and Company (Becton Drive, NJ, United States), and sodium chloride and glucose were obtained from Scharlab (Barcelona, Spain). All these microbiological media were prepared before use by following manufacturer’s instructions. Quality control strain (ATCC 14028) and clinical strains (V08-S-HA-06-170, V15-S-HA-02-210, SAL 109, SAL 202 and SAL 224) of *S.* Typhimurium were used in this study. These clinical strains were obtained from farms of different regions in the Republic of Korea. Clinical isolated strains V08-S-HA-06-170 and SAL 109 were from feces of cattle, V15-S-HA-02-210 and SAL 202 were from feces of chicken and SAL 224 was from carcass of chicken. Isolation, identification and antibiotic sensitivity pattern of these clinical strains were carried out based on previously reported methods [46]. Considering the antibiotic sensitivity patterns, the clinical strains V08-S-HA-06-170 and V15-S-HA-02-210 were characterized as intermediate resistant, and SAL 109, SAL 202 and SAL 224 were identified as resistant. All the strains were cultivated in MHB for 20 h in a rotating incubator at 200 rpm and 37 °C. Antibiotics used in this study include amoxicillin, ampicillin, cefotaxime, ceftiofur, erythromycin, florfenicol, marbofloxacin, norfloxacin, penicillin G and thiamphenicol. GA, epicatechin, epicatechin gallate, epigallocatechin and hamamelitannin were utilized as antibacterial agents in this study. All the chemicals, reagents and media were from Sigma-Aldrich (St. Louis, MO, United States) unless otherwise mentioned. Stock solutions of epicatechin, epicatechin gallate and epigallocatechin were prepared by dissolving in water. Slight heat and sonication were applied to dissolve epicatechin gallate in water. Alcohol was used as co-solvent to dissolve GA and hamamelitannin in preparing stock solutions. All these stock solutions were further diluted to respective media (e.g., MHB, TSB, etc.) before using in experiment. Solvent controls were used where it was required. Respective growth media (e.g., MHB, TSB, etc.) were used as control medium in the combination experiment, unless mentioned otherwise.

### Minimum inhibition concentrations of antibacterial agents

MICs of above mentioned commercial antibiotics and opportunistic antibacterial agents were determined by the standard broth microdilution method according to the clinical and laboratory standard institute (CLSI) guidelines in CA-MHB using an inoculum concentration of 5 × 10^5^ CFU/mL [47]. Different antibacterial solutions were serially diluted in 96-well plates in 100 μL volumes. The starting concentrations of amoxicillin, ampicillin, cefotaxime, ceftiofur, erythromycin, florfenicol, marbofloxacin, norfloxacin, penicillin G, thiamphenicol, epicatechin, epicatechin gallate, epigallocatechin, gallic acid and hamamelitannin against all the tested strains after inoculating bacteria were 512, 1024, 1024, 512, 2048, 512, 32, 32, 1024, 2048, 1024, 512, 1024, 1024 and 2048 μg/mL, respectively. The cultures of different bacterial strains were diluted to adjust 0.5 McFarland units and, again diluted 100-times. Hundred microliter of this diluted bacterial suspension was dispensed to all the wells of 96-well plates which contain 100 μL of antibacterial solution. After incubation at 35 °C for 18 h, the turbidity in each well was checked. The lowest concentrations of these antibacterials that completely inhibited the increase in turbidity were considered as MICs.

### Fractional inhibition concentration index of antibacterial agents

A slightly modified version of the previously described checkerboard microdilution method was utilized to determine the combination interactions of the commercial antibiotics and phenolic compounds [48]. One antibacterial agent was vertically diluted and the other antibacterial was horizontally diluted in 96-well plates to achieve a matrix of different combinations of the 2 antibacterials. Starting concentrations of these antibacterial agents were same as mentioned in ‘Minimum inhibition concentrations of antibacterial agents’ section. Similar dilutions of individual drugs and the drug-free medium control were included in each test plate. *S.* Typhimurium culture in early log phase was diluted and 100 μL of the diluted bacterial suspension was added to each well of the 96-well plates, where the final inoculum concentration after transferring to each well would be 5 × 10^5^ CFU/mL. Plated bacteria were incubated at 35 °C for 18 h. The fractional inhibitory concentration (FIC) and the FICI were calculated from the MICs of the drugs alone and in combination. The FIC is the MIC of a drug in presence of another drug divided by the MIC of the individual drug, and the FICI is the sum of the FICs of the individual drugs. An FICI of ≤0.5 is regarded as synergistic, 0.5 < FICI ≤1 is considered additive, 1 < FICI ≤2 is considered indifferent, and an FICI > 2 is considered antagonistic effects [49].

### Effect of antibacterial combinations on bacterial inhibition rates

The time- and concentration-dependent inhibition effects of GA-ceftiofur combination against *S.* Typhimurium were evaluated according to a previously reported method [14]. Effects of GA (1 × MIC), ceftiofur (1 × MIC), GA (1 × MIC) + ceftiofur (1 × MIC), GA (½ × MIC) + ceftiofur (½ × MIC), and GA (¼ × MIC) + ceftiofur (¼ × MIC) against *S.* Typhimurium were analysed. Drug compounds alone and in combination were supplemented in 10 mL MHB broth in 15 mL falcon tubes. Bacterial cultures in early log phase were diluted and then re-suspended in the drug-supplemented broth to a final inoculum concentration of 5 × 10^5^ CFU/mL. A tube containing 5 × 10^5^ CFU/mL of bacteria in 10 mL MHB without any drug was used as a control. The samples were incubated at 37 °C and 200 rpm in a shaking incubator. At different time points (0, 1, 2, 3, 4, 6, 8, 12, and 24 h) 100 μL of the cultures were collected from all tubes and serially diluted 10-fold in agar saline. Aliquots of the 10-fold dilutions (20 μL) were spread on MHA plates and incubated overnight at 37 °C. The CFUs of the cultures were determined by counting the number of colonies from each dilution. The mean log^10^ CFU/mL for each compound was plotted against different times.

### Effect of antibacterial combinations on bacterial cell morphology

The effects of the GA-ceftiofur combination on the morphology of *S.* Typhimurium cells were evaluated. Drug compounds alone or in combination were supplemented into 10 mL of MHB broth in 15 mL falcon tubes. The concentrations of antibacterials used in this assay are same as mentioned in ‘Effect of antibacterial combinations on bacterial inhibition rates’ section. Bacterial cultures in early log phase were diluted and then re-suspended in the drug-supplemented broth to a final inoculum concentration of 5 × 10^5^ CFU/mL. A tube containing 5 × 10^5^ CFU/mL of bacteria in 10 mL MHB without any drug was used as a control. The bacteria in tubes were incubated overnight at 37 °C and 200 rpm in a shaking incubator. Then, the cells were harvested, washed, and dehydrated according to a previously reported protocol [50]. The ultrastructural morphology of treated *S.* Typhimurium cells was studied using a SEM (models S-4300 and EDX-350; Hitachi, Japan).

### Effect of antibacterial combinations on biofilm growth and viability

The inhibitory effect of combination antibacterials on biofilm formation was determined using slightly modified version of previously reported spectrophotometric methods [51, 52]. Briefly, test compounds were supplemented into TSB in three separate wells of a 96-well microplate for each concentration. The concentrations of antibacterials used in this assay are same as mentioned in ‘Effect of antibacterial combinations on bacterial inhibition rates’ section. Culture of *S.* Typhimurium was incubated for 18 h in a rotating incubator at 200 rpm and 37 °C. The bacterial culture was diluted in TSB, and then 100 μL of the diluted culture was added to the designated wells to a final cell density of 5 × 10^5^ CFU/mL after inoculation. The optical densities (ODs) of the bacteria in the wells of a 96-well plate were measured at 600 nm instantly after inoculating bacteria. The bacteria in the 96-well plate with drugs were incubated for 24 h at 37 °C, and after incubation, the ODs were again measured to determine the growth of planktonic cells. Then the supernatants from the wells of a 96-well plate were discarded carefully without affecting the biofilms which are attached on the well-surfaces. The adherent media and drug components were removed by washing the wells three-times with sterile phosphate buffered saline (PBS, pH 7.2). Then, 200 μL of methanol (99%, v/v) were dispensed to the wells, and kept for 20 min to fix the biofilms. The biofilms were then stained by introducing 100 μL of crystal violet (0.2%, w/v) solution to the wells and keeping at room temperature for 15 min. The excess or unbound crystal violet in the wells was removed by four-times washing with PBS. The crystal violet on the biofilm cells was extracted in 100 μL of 95% ethanol, and their ODs were measured, which yields a measure of biofilm formation (compared to the control). Measurements were performed in triplicate and repeated 3 times.

Slightly modifying previously reported biofilm viability assay method was utilized to evaluate the effects of combination drugs on the viability of the biofilms produced by *S.* Typhimurium [16, 53]. In brief, sterile TSB broth of 2 mL was transferred to a Nunc™ Lab-Tek™ II chambered cover glass (ThermoFisher Scientific, Waltham, MA, United States), and diluted culture of *S.* Typhimurium inoculated into the broth to a final concentration of 5 × 10^5^ CFU/mL. The Nunc™ Lab-Tek™ II chambered cover glasses which contain *S.* Typhimurium cells were kept in an incubator without any agitation at 37 °C until 48 h for biofilm formation. Every 24 h, the TSB broth used in biofilm formation was replaced by fresh, sterile TSB broth without affecting the bacterial cells. The supernatants and planktonic cells were discarded after incubating the bacteria for 48 h, and the chambered cover glasses were washed by 1 × PBS. Then, 2 mL of sterile TSB containing GA (½ × MIC), ceftiofur (½ × MIC), and GA (½ × MIC) + ceftiofur (½ × MIC) were separately added to those chambered cover glasses for treating the biofilms of *S.* Typhimurium. Then biofilm and drug containing chambered cover glasses were again kept in an incubator at 37 °C for 24 h to treat the developed-biofilm cells. After 24 h of exposure to the test compounds, the biofilms were again washed with sterile double distilled water (DDW), and stained with BacLight live/dead stain (ThermoFisher Scientific, Waltham, MA, United States). Untreated biofilm was used as negative control. Positive control could not be used in this study as there is no reliable inhibitor which is proven as well as validated to kill 100% of biofilm cells of this particular strain. Live/dead staining kits have two stains such as SYTO9 and propidium iodide. When used alone, the SYTO9 stain can cross all bacterial cell membranes facilitating a whole cell count [54]. In contrast, propidium iodide penetrates only bacteria with damaged membranes, causing a reduction in the SYTO9 stain fluorescence when both dyes are present [55]. When both dyes are present, propidium iodide exhibits a stronger affinity for nucleic acids than SYTO9, and hence, SYTO9 is displaced by propidium iodide [55]. Thus, the bacterial biofilm cells stained fluorescent green (SYTO9) have intact membranes and considered to be live cells whereas those cells stained red (propidium iodide) have damaged membranes and considered as dead cells. CLSM was used to scan the viable and nonviable biofilms. Imaging was performed with a ZEISS LSM 700 CLSM (Carl Zeiss, Jena, Germany) using 488 nm laser and 495–550 nm emission filter for SYTO9, and 561 nm laser and 560–600 nm emission filter for propidium iodide. ZEN 5.5 software (Carl Zeiss, Jena, Germany) was used to execute image acquisition as well as subsequent image manipulation. Images were captured randomly from different zones of each cover glass. From each zone of a sample, 6 stacks were acquired. Images were analyzed in IMARIS 9.1 software package (Bitplane, Zurich, Switzerland) for the quantification of biofilm biomass. To do so, the original Zeiss files (CZI format) were imported into the software, and determined the biomasses of live biofilm (SYTO9-stained biofilm) and dead biofilm (propidium iodide-stained biofilm) in different observation fields. The biomass of total biofilm in each test group was determined by summing up the biomasses of live biofilm and dead biofilm.

### Effect of antibacterial combinations on the motility of bacterial cells

The swarming and swimming motilities of *S.* Typhimurium (ATCC 14028) in the presence of GA-ceftiofur combination were evaluated according to previously published methods with slight modifications [56]. Nutrient broth supplemented with 0.5% glucose and 0.5% agar was used for the evaluation of *S.* Typhimurium swarming motility. The media used to evaluate the *S.* Typhimurium swimming activity was composed of nutrient broth supplemented with 0.5% glucose and 0.25% agar. Ceftiofur (1 × MIC), GA (1 × MIC), ceftiofur (1 × MIC) + GA (1 × MIC), and ceftiofur (½ × MIC) + GA (½ × MIC) were supplemented in different molten agar plates for determining their effects on swimming and swarming motilities of *S.* Typhimurium. A drug free plate was employed as the negative control. The plates were allowed to dry for 1 h and then 2 μL of *S.* Typhimurium cultures were inoculated onto the respective swarming and swimming agar plates. Inoculated *S.* Typhimurium on swim plates were incubated at 37 °C for 10 h, whereas the bacteria on swarm plates were incubated overnight at 37 °C. After incubation, the swarm and swim zone diameters were measured using calibrated digital slide callipers (Mitotoyo, Japan), and photographs of the plates were captured.

### Cell viability in the presence of antibacterial agents

The in vitro viabilities of *Rattus norvegicus* small intestine (IEC-6; American Type Culture Collection CRL-1592, VA, United States) cell lines in the presence of GA and ceftiofur alone, and GA-ceftiofur combination were evaluated according to standard EZ-cytox (EZ-1000; Daeillab Service Co. Ltd., Jeonju, Republic of Korea) assay method. In brief, IEC-6 cells were cultured at 37 °C under a humidified atmosphere of 5% carbon dioxide (CO_2_) in Dulbecco’s Modified Eagle’s medium (DMEM; ThermoFisher Scientific, Waltham, MA, United States) with 4 mM L-glutamine (ThermoFisher Scientific, Waltham, MA, United States), adjusted to contain 1.5 g/L sodium bicarbonate (Carolina Biological Supply Company, Burlington, NC, United States) and 4.5 g/L glucose and supplemented with 0.1 Unit/mL bovine insulin (90%) and FBS (10%). The cells were sub-passaged at a ratio of 1:5 twice a week. One hundred microliters of suspended cells with the density of 2 × 10^4^ cells/mL were acclimated in 96-well plates at 37 °C under 5% CO_2_ for 24 h. Then, the medium from each well was aspirated and the cells were washed twice. Separately, the test compounds were serially diluted in cell culture medium to make a concentration range as ceftiofur (500–31) μg/mL, and GA (500–31) μg/mL. One hundred microliters of these serially diluted test compounds (GA and ceftiofur alone and their combination) were dispensed into each well which contained washed cells as mentioned above. The cells in the drug-supplemented medium were allowed to incubate for 24 h at 37 °C under 5% CO_2_. A total of 10 μL of EZ-cytox was added to each well. After incubation for 2 h, the absorbance in each well was measured at 450 nm using a plate reader. Cells not treated with any drug was assigned as the control. The cell viability (%) was calculated by the following formula, and values of cell viability (%) at different drug concentrations were used to determine the IC_50_:

Cell viability (%) = (OD of drug-treated sample/OD of untreated sample) × 100, where OD is the optical density [57].

### Statistical analysis

Results are presented as the means ± standard deviation (SD) of triplicate analysis. Statistical analysis was carried out by using SAS software (SAS Institute Inc., Cary, NC, United States). One-way analysis of variance (ANOVA) followed by F-test was used to compare the results. Statistical significance was considered when the *p*-value was < 0.05.

## Supplementary information

**Additional file 1. **Effects of gallic acid alone and in combination with ceftiofur on the viability of *Rattus norvegicus* small intestine (IEC-6) cells.

## Data Availability

Data will be shared upon request to the corresponding author.

## Abbreviations

CA-MHB: Cation-adjusted mueller hinton broth
CLSI: Clinical and laboratory standard institute
CLSM: Confocal laser scanning microscope
DDW: Double-distilled water
DMEM: Dulbecco’s modified eagle’s medium
FIC: Fractional inhibitory concentration
FICI: Fractional inhibitory concentration index
GA: Gallic acid
IC_50_: Inhibitory concentration 50%
LB: Luria-bertani
MHA: Mueller hinton agar
MHB: Mueller hinton broth
MIC: Minimum inhibitory concentration
NB: Ntrient broth
NTME: *Nymphaea tetragona* 50% methanol extract
OD: Optical density
PBP: Penicillin-binding proteins
PBS: Phosphate buffered saline
QC: Quality control
QS: Quorum sensing
SEM: Scanning electronic microscope
TSB: Trypticase soy broth
