# Supplementary material for: In vitro synergistic potentials of novel antibacterial combination therapies against Salmonella enterica serovar Typhimurium
Source: BMC Microbiol. 2020 May 14;20:118. doi: 10.1186/s12866-020-01810-x (PMC7227263; doi:10.1186/s12866-020-01810-x)
Supplement: Supplementary file 1 — Additional file 1. Effects of gallic acid alone and in combination with ceftiofur on the viability of Rattus norvegicus small intestine (IEC-6) cells. [file 12866_2020_1810_MOESM1_ESM.docx]

**Additional file 1:** Effects of gallic acid alone and in combination with ceftiofur on the viability of *Rattus norvegicus* small intestine (IEC-6) cells.

| **Antibacterials** | **Inhibitory Concentration 50% (µM)** |
| --- | --- |
| Ceftiofur | 694.05 |
| Gallic acid | 564.55 |
| Gallic acid + (60-955) µM Ceftiofur | 1907.55 |
| Ceftiofur + (184-2939) µM Gallic acid | 604.83 |

Results are interpreted from 3 independent experiments.
